# Research on the temporal evolution track and influence of green development from 2010 to 2019

**DOI:** 10.1371/journal.pone.0263482

**Published:** 2022-11-14

**Authors:** Yunfang Chen, Xuan Ji, Qingkui Lai

**Affiliations:** 1 Faculty of Geography, Yunnan Normal University, Kunming, China; 2 Institute of International River and Eco-Security, Yunnan University, Kunming, China; 3 Economics and Management School, Southwest Forestry University, Kunming, China; Sichuan Agricultural University, CHINA

## Abstract

This study aims to analyze the balance and coordination during green and sustainable development and estimates the evolution track of the green development system from 2010–2019. The projection pursuit model (PPM), as well as the system gray prediction model GM (1, N) and other measurement methods, were used to construct an evaluation system accounting for green fortune, growth, and benefits to analyze the temporal evolution, developmental trends, and influencing factors of the ecological engineering construction area in Baoshan, Yunnan Province. The results revealed a relatively good green development system, with an upward trend, an average growth rate of 18.3%, and a synergistic coupling effect among the three subsystems. Regional green development has achieved remarkable progress, but challenges and pressure among the three subsystems remain. The prediction analysis also showed that the green development index in Baoshan will continue to increase over the next two years, suggesting that the regional green development system is effective. Green development is primarily affected by environmental pollution, industrial structure, urbanization, population, market, and other factors from the three subsystems of ecology, economy, and social benefits. This study provides practical reference values for prompting regional ecological advancement and green development, along with regional support for the post-2020 Global Biodiversity Framework and the achievement of the United Nations Sustainable Development Goals.

## Introduction

In the 17th century, Adam Smith proposed that the increase in world population would trigger a shortage in natural resources; the resources were scarce and the future human civilization would experience a contradiction between population growth, limited land, and food demand [1]. In 1962, biologist Rachel Carson illustrated the destructive effect of industrial development on the ecological environment in her book “Silent Spring,” arguing that economic development should simultaneously focus on protecting the ecological environment. The idea of green development has originated from sustainable development, green economy, and low-carbon economy [2]. In 1987, the World Commission on Environment and Development formally presented the concept of sustainable development in the working report “Our Common Future,” indicating that green development is essential to realizing the concept of sustainable development recognized by the international community. The British environmental economist Pearce’s “Blueprint for a Green Economy” first proposed a “green economy” and advocated for the establishment of an economic development method with affordable resources and an environment-friendly industry [3], and further proposed a low-carbon economic development approach. In 2003, the “low-carbon economy” concept was formally introduced in the British government’s energy white paper. In 2005, the Organization for Economic Cooperation and Development (OECD) defined the concept of “green growth” in response to the contradictory issue of environmental development as the promotion of economic growth and development while preserving natural resources. In 2008, the United Nations Environment Programmer introduced the Global Green New Deal [4], which triggered a green economic paradigm shift. The G20 summit in 2015 put forward the concept of “insisting on green and low-carbon development.” The G20 Germany summit in 2017 released the “2017 G20 Green Finance Synthesis Report,” which focused on analyzing the current challenges of green finance from environmental risks and public environmental data. The European Union, the United States, Japan, and other developed countries also formulated a series of strategic plans with green manufacturing and green energy technologies, actively implementing the Green New Deal and opening a new era of the green industrial revolution to achieve economic recovery through the development of the green economy and green industry, taking an advantageous position in international competition.

China is currently afflicted by severe resource and environmental pressure; in addition, population, resource, and environmental conditions necessitate the implementation of a sustainable development strategy. The White Paper on Chinese Population, Environment, and Development in the 21^st^ Century is the first sustainable development strategy in China. The national 10^th^ and 11^th^ Five-Year plans promoted the constant implementation of the strategy and appealed to developing a resource-conserving and environment-friendly society. Green development has been suggested in the 12^th^ Five-Year Plan, advocating for “establishing the concept of green and low-carbon development.” The 13^th^ Five-Year-Plan suggested that greening practices are necessary for sustainable development and is an important embodiment of the pursuit of a better life.

The report to the 19^th^ National Congress of the Communist Party of China (CPC), highlighted the need for accelerating the reform of the ecological civilization system to better meet the growing ecological needs of its citizens; it emphasizes the strategic development direction of realizing an excellent ecological environment through green development. To date, China’s green development has gradually become more complex with multi-dimensional exploration.

Scholars have had many theoretical explorations on the concept and connotation of green development, while dynamic and static assessment, driving forces, and realization approaches have also been explored. Nonetheless, major issues remain and must be addressed. First, the concept of green development in China is diversified but also limited because of the combination of theory and practice from multiple disciplines and perspectives. Second, green development implies ecological protection, which is integrated with the coordinated development of the economy and society. However, the integrated evaluation of economic and social development leads to an unclear assessment of green development; a systematic and overall understanding of China’s green development is lacking, which leads to the failure in understanding green development from the perspective of green economy, industrial greening or ecological systems, resources, and energy systems. Green development has gradually become the inevitable choice for many countries to confront financial crises, environmental and resource challenges, and sustainable development. Based on understanding the green development system and its connotations, this paper introduces an evaluation system for regional green development.

**Fig 1:**
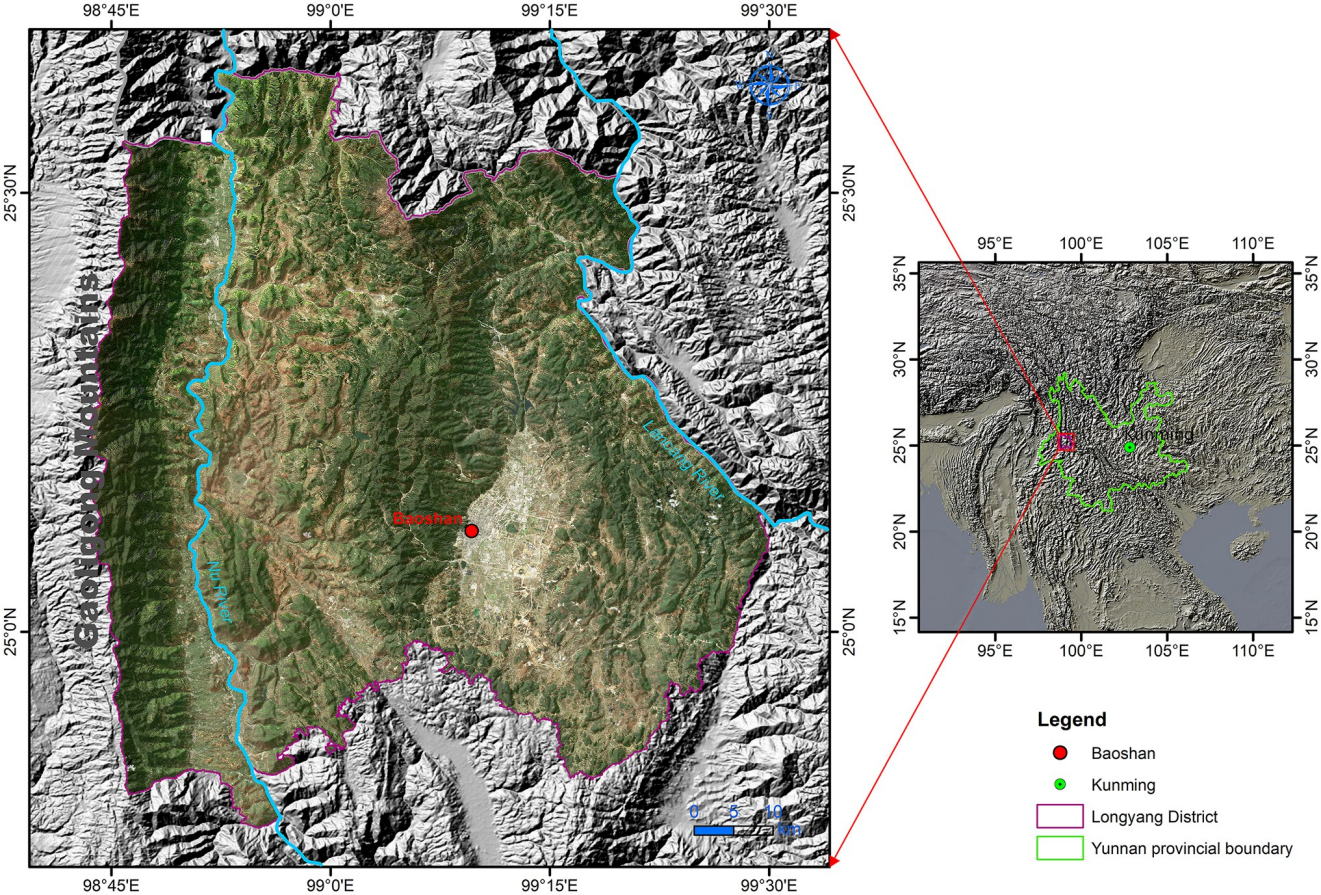
Maps of the study area. The satellite image is Sentinel-2 data obtained from Copernicus Open Access Hub, with permission from the European Space Agency (ESA; https://scihub.copernicus.eu/dhus/#/home). Credits: Contains modified Copernicus Sentinel data [2019], processed by ESA. The terrain information in the map was derived from the Shuttle Radar Topography Mission (SRTM) DEM, which is available from NASA (https://earthdata.nasa.gov/). The figure was made with ArcGIS 10.3 under a CC BY license, with permission from ESRI (www.esri.com).

In Fig 1, Baoshan is in the core area of the Gaoligong Mountain National Protected Area, which is rich in biodiversity and has the most complete ecosystem protection. Longyang District under its jurisdiction has a forest coverage rate of 65.56% and a unique and superior climate environment described as “four seasons in one mountain and different weather every ten miles.” As a mountainous and multi-ethnic area, Baoshan has been exploring a method of green development under the premise of environmental protection. The livelihood of residents and the development of regional production are highly dependent on forest ecosystems and ecological products, such as forest recreation, coffee planting, and ecological tourism services. It is an ecological engineering and green industry demonstration area of “eating organic food, breathing fresh air, enjoying the beauty of forests and natural landscape;” therefore, green development is a sustainable method for Baoshan to balance economic and social development with ecological protection.

Following the development direction of the Three Green Cards of the Yunnan Government in 2016, Baoshan inaugurated the ecological engineering project in combination with its regional characteristics and urban ecological developmental strategy to integrate the elements of “mountain, water, and city” and develop a new blueprint for regional ecological development. Implementing the ecological engineering project will not only promote the development of an ecological ethos in Yunnan but also improve the living standards of its residents and benefit the economic development of surrounding areas. Hence, this paper assesses the changes in the green development index, level, and the evolutionary paths promoting the ecological engineering project over the past ten years in the Longyang District, Baoshan, and provides a reference for the promotion of green development and an ecological civilization construction in the Yunnan Province, China.

This study focuses on the existing green development model in Baoshan and constructs an evaluation system, including three dimensions of green wealth, green increase, and green welfare, to analyze the temporal evolution, developmental trends, and influencing factors. This study makes a significant contribution to the literature because it investigates an existing green development model in China and assesses its success and possible improvements, suggesting models that can be implemented to advance green development and the symbiotic existence between humans and the environment.

## Literature review

Green development is a new sustainable development model based on the traditional linear and rough development model, which promotes the coordinated development of socio-economic and environmental resources through the effective utilization of resources, clean production, and improvement of sustainable development methods [5]. Green development originated in the United Nations Development Program’s China Human Development Report 2002: Green development is the important way to sustainable development. The report to the 19^th^ CPC National Congress proposed that building a beautiful China requires actively promoting green development to address environmental pollution and ecosystem degradation attributed to the rapid economic growth.

Research on green development involves various disciplines [6] such as green economic measurement [7], green development efficiency [8, 9], green supply chains [10], green policies and institutions, green buildings [11, 12], as well as comprehensive measurement and impact factors [13]. Chinese research on green development has focused on the main aspects of concepts and methods, research content, and research space, mainly including entropy-TOPSIS [14], projection tracing models [15, 16], principal component analysis, envelope analysis [17], dissipative structure principle, and the comprehensive weighting method [18]. The research targets green development connotation, theory [19], urban green development system [20] (such as the system of the green infrastructure, which is the study of networks connected by natural areas and other open spaces [21]), green development level assessment and calculation [1], spatial change characteristics of green development [22, 23], green efficiency and green technology [24, 25], and green influence factors analysis [15], to measure urban land use efficiency [26]. The spatial aspect of the study covers the evaluation of green development at different geographical scales, such as national [27], provincial, municipal [28, 29], and special regions [14]. Based on the green development system under the complex system theory, this study focuses on the composition of complex ecological, economic, and social adaptive systems [30] of regional green development. An evaluation, measurement, and prediction with a certain practical reference value were conducted to promote the establishment of a regional ecological civilization and green development. As shown in Table 1, it has summarized the main research directions of current green development.

**Table 1:** Literature review of green development research.

Research time	Last name of first authors	Scope	Result/Discussion/Key-findings
1999–2017 2005–2014	Li Mu	Theoretical framework and formation of Green development	Green development is a complex adaptive system related to the ecological, economic, and social subsystems. The green economic development and differentiation in resources, environment, and regional policies are the main factors driving regional differences in green production efficiency.
2000–2015	Li	Review research	Analyzed and summarized the research hotspots, frontiers, knowledge base, and the spatiotemporal distribution of Chinese and international scholars of green development. The frontiers of China’s green development research focus on coordinated development, ecological environment, and green development concepts.
2007–2017 2016 1995–2015	Du Halkos Cabernard	Green economy/mechanism of high-quality development	The study reports a “resource curse” in the stage of high-quality economic development at the provincial level in China. These authors demonstrated that far more action is needed to move toward a greener economy globally, especially through supply chain management, innovation investment, and talents that wish to transform and upgrade the resource-based regional economy. In addition, Halkos et al. have analyzed 83 economies concluding that industrialized economies outperform other rivaling economies. The analysis offers avenues for supporting novel approaches to the structural transformation of economies.
1990–2014	Sun	Green technology	The study evaluated and examined the impact of both governmental institutions and green technologies on energy efficiency. It highlighted the significant positive influence of green innovation and institutional quality on energy efficiency.
2005–2015 2005–2015	Zhou Che	Green development efficiency	These researchers found that China generally displays an EKC effect; environmental pollution continues to increase while the economy is developing. The degree of urban green development efficiency reflects the quality of the natural environment and the level of urban environmental regulation.
1998–2017	Tseng	Green supply chain	Research on drivers revealed a declining trend of barrier analysis of green supply chain management while mathematical optimization models are increasingly being applied to enhance decision-making in pursuit of environmental performance.
2020 2008–2018	Gan Gao	Green buildings	A review of computer simulation and optimization studies for minimizing the life cycle energy consumption and carbon emissions in buildings aimed to identify current practices and future research needs in this field. The analysis focused on the development status of China’s green buildings from three perspectives: equilibrium, spatial distribution characteristics, and spatial correlation.
2005–2019 2009–2018 2005–2016 2000–2016	Lu Peng Guo Cheng	Comprehensive measurement of green development	Used the PPM as a measurement method to determine the impact of financial development on ecology and green development. The study suggested enterprises should strengthen technological innovation to improve resource use efficiency and reduce pollution by continuously improving the green development index system.
2015	Wei	Green infrastructure	It demonstrated the relationship between the spatial morphology characteristic of green infrastructure. It played a key role in fostering green development.
2017 2000–2018	Gao Zhao	Green Network	It constructed a city-scale apace system and evaluated the importance of the landscape elements of green space. The green efficiency network had a significant spatial correlation and spillover effect among provinces, which proved significant for the construction of urban green space.
2009–2018 2003–2017 2013–2016 2000–2015 2000–2015	Zhang Tang Wang Zhao Hou	Spatial evolution of green development	The level of green development in China reflects the level of economic development, industrial structure innovation input, urban expansion, land use changes, and influencing factors. The social benefits and undesired outputs brought about by urban development are considered based on emphasizing economic output.

This study was primarily based on the complex system theory; calculations were used to estimate the regional green development index and assess the temporal evolution of green development. Our study considered green development to be composed of three subsystems: green wealth, green increase, and green welfare, assessing the relationship between the development of the three subsystems. Challenges were identified through econometric analysis of regional green development, and solutions and countermeasures were proposed.

### Measurement methods of the green development index

#### The basic framework of green development evaluation

The essence of green development is realizing the concept of sustainable development as recognized by the international community [31]. The relationship between green development and sustainable development is the same; green development is not only the inheritance of sustainable development but also the theoretical innovation of sustainable development in China. It is also a significant theoretical contribution to China’s response to global environmental deterioration. Green development refers not only to the ecological environment and sustainable construction, but to an organic combination of the three subsystems of natural ecology, economy, and society. Green development is a high-efficiency path yielded by coordinating ecology, the economy, and society with systematic integrity and dynamism, aiming to enhance human welfare and accumulate green wealth. Green development is accompanied by economic behaviors of low and reasonable consumption and low emission. The main goal of green development is the continuous enrichment of ecological capital to achieve harmonious coexistence and mutual benefit between humans and nature. Green development is required for sustainable development, an important manifestation of Chinese citizens’ pursuit of a better life, and a fundamental approach to Chinese ecological civilization.

The meaning of green development varies but has the same connotation in academia; that is, focusing on the process of high-quality regional development in terms of development efficiency and innovation-driven process [32] while maximizing ecological, economic, and social benefits [33, 34]. Therefore, the optimal development of coordinated regional resources, as well as environmental, economic, and social systems, is an important step towards realizing green development.

Respect for ecological, economic, and social systems is the requirement for green development, which covers three areas: first, to realize the green restructuring of resource elements allocation and promote the transition of green production function; second, to realize the decoupling of economic growth and development speed from an ecological deficit; and third, to establish the development concept of “unity of nature and man” and mutual benefit of nature and humans [2].

From a system perspective and based on system function and mechanism, strategies for green development were analyzed. The Three Circles model was set up, with green development regarded as the second generation of sustainable development, including green wealth, growth, and benefits. Among them, green wealth is a foundation, green growth is the means, and green welfare is the target [2]. Therefore, green development is a relief-efficient type of sustainable development in coordination with the economic and social development of an efficient symbiotic environment with a developmental direction. This is achieved by realizing a resource-saving transformation within the regional system rendering the industry environmentally friendly, improving clean production strategies, and restructuring the production process [4] to achieve green and high-level development of ecological civilization in the region.

#### Indicator system of green development

With the extensive application of green economy and sustainable development in recent years, the evaluation index system has been continuously developed, including mainly environmental sustainability, inclusiveness, and green economy.

Research on the evaluation of green development mainly covers two aspects: the evaluation and construction of the green development measurement index system. Methods of green development evaluation include input–output and cost–benefit models, entropy methods [35], spatial autocorrelation analysis [15], LMDI measurement models [36], and environmental carrying capacity. In terms of indicator selection and system construction of green development, most indicator systems are selected by referring to the environment carrying capacity index, green industry, and green economy evaluation, or the green GDP of resource and environmental cost and ecological efficiency index calculated to measure the efficiency of green development [37, 38]. For example, a green development index was established based on the human development index and was measured in 123 countries and regions [39]. One study analyzed the change in the time pattern of the green economy by calculating China’s green GDP [40]. Scholars have also studied the spatial evaluation model of China’s green development efficiency from the perspective of resources and the environment using data envelopment analysis [41].

As for the green development index, Yang Duogui [42] constructed an evaluation system by analyzing environmental benefits, energy consumption, environmental metabolism, environmental pollution, and resource consumption. The green economy development index is also established according to the principle of ecosystem material flow, containing three aspects; namely, green production, consumption, and health [43]; besides, based on green production, living, green environment, and new green policies, studies have constructed an evaluation index system of green development at the provincial level [31].

Previous research on green development in China focused on the evaluation of the green economy, industrial greening degree, and sustainable development degree necessary to conduct a systematic assessment of the current situation and deficiency of green development and conduct a systematic evaluation, condition simulation, and development trajectory analysis of green development.

Based on the green development concept put forward by Hu Angang, this study constructed the evaluation index system of green development according to its green wealth, increase, and welfare. Considering the current research results and regional green development strategy design, the regional green development evaluation index system was designed according to the scientific, regional, operable, and comprehensive systematic principles [28]. This study analyzes the green development evaluation system, including three layers: the first layer for the total system; namely, the development of the regional green evaluation index system; the second is the subsystem layer or the evaluation layer of ecological economic and social subsystem; the third layer includes green wealth, growth, and welfare. Overall, the three-layer index contains a total of 25 indicators. Among them, green wealth includes seven basic indicators such as annual afforestation area, green coverage rate, per capita arable land, and regional emission of SO_2_; the green increase covers eight basic indicators, including per capita GDP, energy consumption, and land productivity; green welfare includes ten basic indicators such as the number of students in this area, the area’s average income, per capita public green land, and effective irrigation area (Table 2 below).

**Table 2:** Construction of regional green development.

System	Subsystem	Basic indicators	Attribute
The Regional Green Development	Green Wealth	Annual afforestation area (hm^2^)	+
		Green coverage of urban area (%)	+
		Arable land per capita (hm^2^)	+
		SO2 emissions (1,000 tons)	-
		Industrial effluent discharge (1,000 tons)	-
		Consumption of N/P/K chemical fertilizers (ton)	-
		COD emissions (1,000 tons)	-
	Green Increase	GDP per capita (CNY)	+
		Energy consumption (t ce)	-
		Annual output value of primary industry (10,000 yuan)	+
		Land productivity (%)	+
		Annual output value of secondary industry (10,000 yuan)	+
		Annual output value of tertiary industry (10,000 yuan)	+
		Gross tourism revenue (10,000 yuan)	+
		The proportion of consumption residential energy	-
		Regional population (10,000 people)	+
	Green Welfare	Students enrollment (10,000 people)	+
		Annual per capita disposable income of urban residents (yuan)	+
		Number of buses	+
		Rural per capita net income (yuan)	+
		Per capita share of public green space (m^2^)	+
		Urban green land rate (%)	+
		Electricity for rural use (kW·h)	-
		Effective irrigation area (hm^2^)	+
		Urban population (10,000 people)	+

#### Method of regional green development index measurement

The regional green development index was evaluated with the projection pursuit model (PPM) [16], which systematically processes the nonlinear and multi-dimensional data and solves the multivariate complex system evaluation problem, effectively calculating the regional green development. Using the projection optimization function to reduce the dimensions of the multi-dimension data and the low-dimensional data to simulate and analyze the characteristics of the multi-dimensional data [15], the following formulas were applied:

1. Data standardization
$$\text{Positive}:\;\text{x}\;\left(\text{i},\;\text{j}\right)=[\text{x}\left(\text{i},\;\text{j}\right)–{\text{x}}_{\text{min}}\left(\text{j}\right)]\;/\;[{\text{x}}_{\text{max}}\left(\text{j}\right)–{\text{x}}_{\text{min}}\left(\text{j}\right)]$$
$$\text{Negative}:\;\text{x}\;\left(\text{i},\;\text{j}\right)=[{\text{x}}_{\text{max}}\left(\text{j}\right)–\text{x}\;\left(\text{i},\;\text{j}\right)]\;/\;[{\text{x}}_{\text{max}}\left(\text{j}\right)–{\text{x}}_{\text{min}}\left(\text{j}\right)]$$
2. Projection function
  If n = {n (1), n(2), …, n(q)} is the projection direction vector, and the one-dimensional projection value of region i:
$$\text{Z}\left(\text{i}\right)=\sum_{\text{j}=1}^{\text{q}}\text{n}\left(\text{j}\right)∙\text{x}\left(\text{i},\text{j}\right),\;\text{i}=1,2\cdots ,\text{m}$$
  The project objective function:
$$\text{Q}\left(\text{m}\right)={\text{S}}_{\text{z}}∙{\text{D}}_{\text{z}}$$
  where Sz is the standard deviation of Z(i); Dz is the local density of Z(i).
$${\text{S}}_{\text{Z}}=\sqrt{\sum_{\text{i}=1}^{\text{m}}{\left(\text{Z}\left(\text{i}\right)-\text{E}\left(\text{z}\right)\right)}^{2}}/\sqrt{\left(\text{m}-1\right)}$$
3. Optimization of the projection objective function
$$\sum_{\text{j}=1}^{\text{q}}{\text{n}}^{2}(\text{j}=1)$$
  The optimization gist of the projection is a nonlinear optimization problem with the variable: {n(j)/j = 1, 2, …, q}; the maximum value can be processed using an accelerated genetic algorithm [15]. Parameters were as follows: initial scale parameter 400, crossover probability 0.8, accelerate times: 50.
4. The optimal value. The optimal projection direction (i.e., weight) n* is multiplied by the normalized index value; the projection value X(i) is calculated as the sum of the evaluation scores of the green development index. The larger the projection, the higher the score:
$$\text{X}\left(\text{i}\right)=\sum_{\text{j}=1}^{\text{q}}{\text{n}}^{*}∙\text{x}(\text{I},\text{j})$$

#### The main source of indicator data

Based on the above evaluation index system of regional green development, the study considered Baoshan, a project area of 10,000 mu, as the research object; the basic index was derived from the Statistical Yearbook of Baoshan City, Yunnan, Statistical Yearbook of Longyang District, Baoshan City, Yunnan, and the environmental statistical data of the study area from 2010–2019.

### Analysis of the evolution track of green development

#### Temporal evolution track analysis of green development

**Fig 2:**
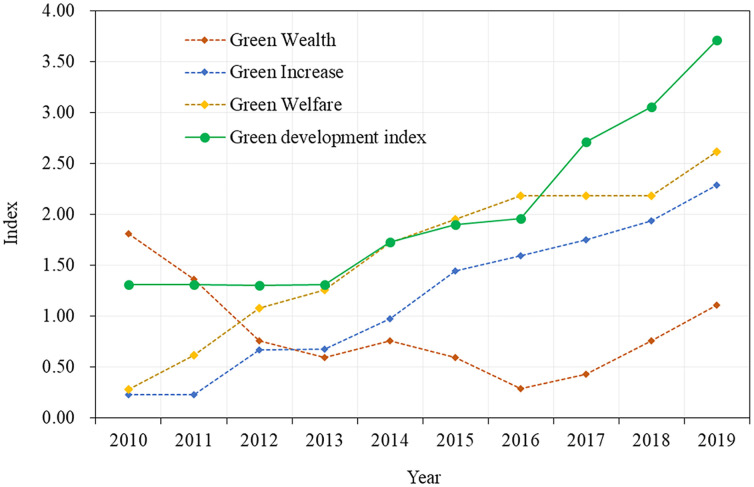
Green-development index of ecological engineering of project area in Baoshan.

The PPM was used to assess the green development validity of the 10.000 mu project of Baoshan in the Yunnan Province (Table 3). From the perspective of the overall development system of this region, the overall green development level shows an increasing trend, from 1.307 in 2010 to 3.708 in 2019, the average annual growth rate was 18.3%, indicating that the level of green development in this region has significantly improved, as shown in Fig 2. Realizing the substantial effect of the resource environment on the regional economic and social development, the local government must follow the natural ecology of the regional development pattern and economic laws for improving green development as a higher developmental strategy, focusing on the coordinated development of the local ecological environment, economy, and society to optimize the sustainable development of regional human and land systems. The government of Baoshan has made a great effort to construct an ecological corridor project covering an area of more than 10,000 mu, actively promoting the building of new urban structures and rural revitalization of the demonstration zone, thereby promoting regional crossways development and accelerating urban ecological development and the sustainable development of local production, consumption structure (with the system structure being constantly optimized), strengthening urban environmental governance, reducing resource consumption, and improving the level of ecological progress. However, the evaluation of the local green development system showed that the overall growth rate of green development in China is slow [15, 47], and the concept of sustainable development must be internalized into the comprehensive decision-making of local top-level design, planning, and development.

**Table 3:** Study area development index and its composition from 2010–2019.

Year	Green Wealth	Green Increase	Green Welfare	Green-Development index
2010	1.808	0.232	0.283	1.307
2011	1.359	0.232	0.618	1.307
2012	0.759	0.673	1.078	1.305
2013	0.593	0.675	1.260	1.310
2014	0.759	0.973	1.721	1.727
2015	0.593	1.441	1.949	1.900
2016	0.292	1.595	2.186	1.962
2017	0.434	1.749	2.186	2.709
2018	0.759	1.935	2.186	3.056
2019	1.109	2.284	2.614	3.708

Based on the structure of the green development assessment system in the construction area, the “green wealth” subsystem was in a declining state at this stage. Fig 2 shows that the “green wealth” index, which represents the regional ecosystem and environmental pressure from 2010 to 2016, continued to decline significantly from 1.808 in 2010 to 0.292 in 2016, with an annual decline rate of 12% (Table 3). Therefore, via restoration of wetlands and strengthening of ecological management, Baoshan has implemented the “Three Ten thousand mu Project” since 2016, that is, the 10,000 mu wetland restoration project, the 10,000 mu sightseeing agriculture restoration project, and the 10,000 mu ecological management vegetation restoration project in Dongshan, integrating the elements of mountains, water, fields, parks, and towns to promote the ecological development of regional cities. The development index of the green wealth subsystem in the construction area of 10,000 mu has significantly recovered from 2017 to 2019, increasing from 0.292 in 2016 to 1.109 in 2019 (Table 3). Evidently, during the study period, local governments actively responded to national policies, strengthened environmental governance investment and management, and promoted the green industrial transformation and ecological red line strategy, so that the uncontrolled development and utilization of regional resources and environment and the disorderly expansion of land space gradually came under effective control. The discharge of SO_2_, industrial wastewater, the application of nitrogen, phosphorus, potassium, and compound fertilizer, and the discharge of COD in the study area have been reduced. In contrast, the ecological and environmental functions of the region have been significantly restored, indicating that the resource consumption intensity, pollutant discharge, and economic scale growth of the region slowly and gradually decoupled.

With the continuous improvement of the regional economic developmental, economic subsystem, the involvement of the structures in the direction of rationalization and degree of order of the green industry economy also gradually improved, the subsystem of “green increase” representing economic development and the subsystem of “green welfare” representing social development elements exhibited a continuous and steady growth trend. The “green growth” index significantly increased from 0.232 in 2010 to 2.284 in 2019. The development index of the “green welfare” subsystem increased from 0.283 in 2010 to 2.614 in 2019, showing significant growth, which corresponds to the improvement of economic development with local public services and residents’ social welfare (Table 3 and Fig 2). This indicates that during the research period, intensive production, as well as recycling at the local level have improved; however, because of the contradictions between the increasing resource load, the environment, and the demand for continuous economic development [1, 28], the development of the “green wealth” subsystem, which represents the economy, is relatively slow, reflecting the difficulties and long-term nature of the sustainable development task of the regional ecological environment system.

The local government has promoted urbanization and rural revitalization of the demonstration area and the central border cities of western Yunnan to promote the production and consumption structure, and optimize the elements of sustainable development integration, strengthen local environmental pollution control, gradually reduce the resource consumption, and improve the level of regional ecological civilization construction.

In addition, the current level of green development in the region must be further improved, while the concept of sustainable development must be internalized and integrated into the local government’s decision-making and development planning and design.

#### Prediction and calibration of green development index in construction areas

The model GM (1,1) describes the prediction model of the variation trend correlation degree of the regional green development index in the short term [44]. GM (1,1) contains only one parameter; hence it has greater flexibility and wider applicability for measuring the sample number and sequence length, and good prediction accuracy for local green development systems. The multi-index complex system of green development as a single comprehensive index system evaluation using the PPM method, which is substituted into GM (1,1) for model prediction, and the green development trend of the research area in the short term is measured. The Precision grade of this model is listed in Table 4.

**Table 4:** Precision grade of gray model GM (1, 1).

GM Accuracy-Grade	Small Error-Probability	Posterior Ratio
Level 1-good	P≥0.95	C≤0.35
Level 2-qualified	0.80≤p<0.95	0.35<C≤0.50
Level 3-fair	0.70≤p<0.80	0.50<C≤0.65
Level 4-unqualified	p<0.70	C>0.65

Taking the area as the research object, the characteristic value of the green development index (2010–2018) was obtained using the above PPM model. Evaluation and analysis were performed with the gray model GM (1,1) to predict and evaluate the change in the regional green development level in the short term (Table 5).

**Table 5:** Dynamic evaluation and forecast results of green development of Baoshan city, Yunnan Province from 2010 to 2018.

Year	2010	2011	2012	2013	2014	2015	2016	2017	2018
Original sequence	1.307464273	1.307464209	1.305316214	1.309872736	1.727271031	1.899596651	1.962202329	2.708768395	3.05585641
Simulated sequence	1.307464273	2.396565018	3.652078832	5.099433396	6.767941709	8.691395753	10.90875088	13.46491476	16.4116569
GM predictive value	1.307464273	1.089100746	1.255513813	1.4473545642	1.668508313	1.9234540450	2.2173551275	2.556163884	2.946742143
Relative error %	0.00	-16.7013	-3.81535	10.49582	-3.40206	1.255919	13.00339	-5.63372	-3.57066

By substituting A = [1.307464273 1.307464209 1.305316214 1.309872736 1.727271031 1.899596651 1.962202329 2.708768395 3.055856406] in GM (1,1), as the initial series x (0), we obtain the response function:

$$\text{F}\left(\text{k}\right)=\left(\text{A}\left(1\right)–\text{u}/\text{a}\right)/\text{exp}\left(\text{a}\cdot \;\left(\text{k}–\;1\right)\right)\;+\text{u}/\text{a},\text{X}\left(\text{k}\right)=\left(1.3075\;–\;0.8276\;/\;\left(-0.1422\right)\right)\;/\text{exp}(\left(-0.1422\right)\cdot \left(\text{k}-1\right))\;+\;0.8276\;/\;\left(-0.1422\right)$$

That is

$$\text{X}\left(\text{k}\right)=7.1275/\text{exp}(\left(-0.1422\right)\cdot \left(\text{k}–1\right))–5.82,$$

Thus, the simulation data sequence is generated, and the prediction sequence is obtained through reduction calculation, as shown in Table 5.

**Fig 3:**
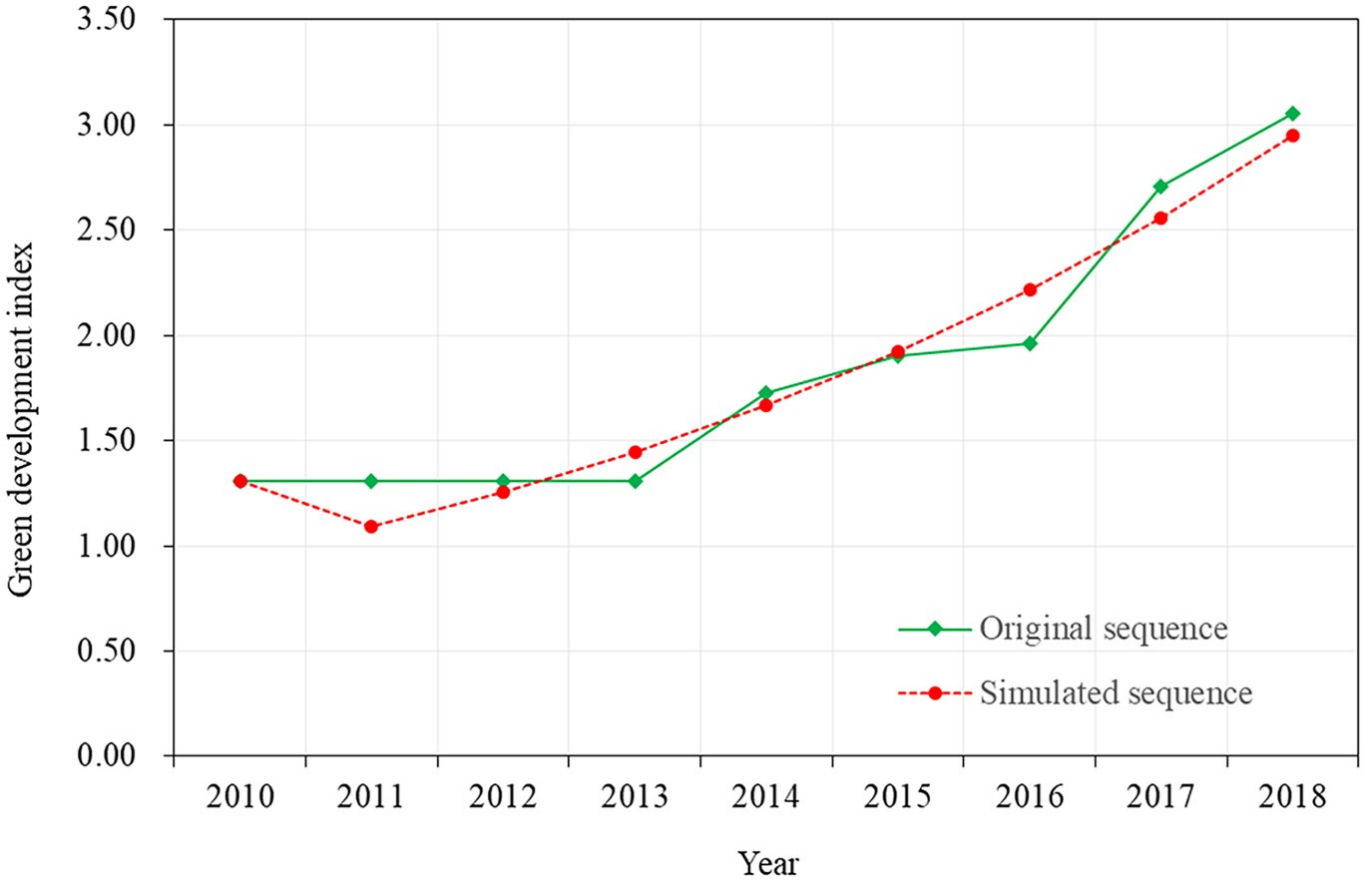
Dynamic simulation analysis of green development of the Baoshan ecological engineering area from 2010 to 2018.

Based on Table 4, this study used the projected eigenvalue 3.708 (Table 3) of the green development index in 2019 as the verification group and substituted it into the prediction model GM (1,1) [44] to calculate the simulated predicted value of 3.398266. After comparing the actual value (the projected eigenvalue 3.708), the relative error was -8.364%. The small error-probability was P = 100% > 95%, the posterior ratio was C = 0.0501 < 0.35, the average absolute value of relative error was -0.9298% < 10%, R2 was 0.95, and the prediction accuracy of the model was level 1. Through the calculation process and the posterior test of the prediction model, from 2020 to 2021, the characteristic value of green development in the Baoshan 10,000 mu project area in Yunnan Province was 3.916436 and 4.514896, with an estimated annual growth rate of 15.1%, indicating steady growth (Fig 3).

### Analysis of the influencing factors of regional green development

This study analyzed the influencing factors between the regional green development system and its constituent subsystem index, combined with the situation and problems faced by the comprehensive sustainable development in Baoshan. The interaction and evolution between the green development composite system and subsystems, which were mainly affected by various environmental conditions such as internal and external conditions, were also analyzed [24, 30]. The resulting measurements showed that the local green development index and its composition were mainly affected by environmental pollution, industrial structure, urbanization, population, market, and other factors from the three subsystems of ecology, economy, and social welfare.

#### Regional eco-environmental factors and green development

**Fig 4:**
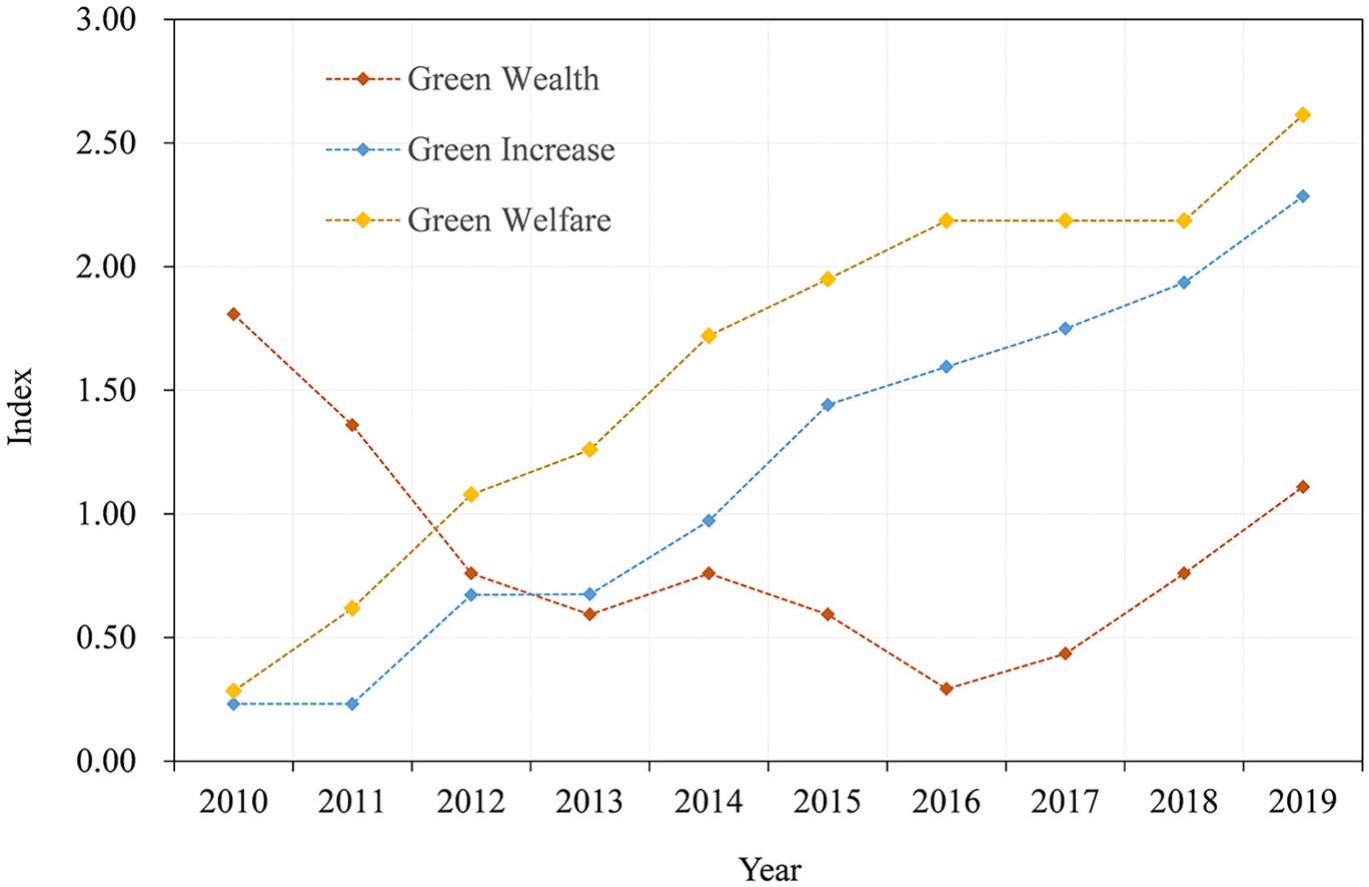
Subsystem index of green development of the Baoshan ecological engineering area.

As shown in Fig 4, applying the PPM model to process the “green wealth” parameters representing the regional ecosystem subsystem revealed a maximum projection value of 0.455. Therefore, the closer to the maximum projection values, that is, the parameters with relatively greater impact on the green development of the green wealth subsystem, were in the following order: “NPK and compound fertilizer (chemical fertilizer) application amount *(0.56)”, annual afforestation area (0.53), industrial wastewater emission *(0.47), and SO_2_ emission *(0.42), among which “annual afforestation area” was positively correlated with the development and evolution of the green wealth subsystem, while other parameters were inversely correlated. The green wealth subsystem dropped from the peak value of 1.81 in 2010 to the minimum value of 0.29 in 2016, with a significant decline in developmental trend and a slow recovery since 2017. With the continuous promotion of local urbanization, the demand for green development and the concept of ecological civilization have gradually strengthened; demands for a good ecological environment also promoted the local government to improve the ecological environment governance system. For example, the annual afforestation area has increased, while the environmental pollution sources have been well controlled [14, 16]. In contrast, under a sufficient supply of environmental, financial funds, a large area of demolition work was conducted in Baoshan from 2013 to 2017, inevitably producing vacant land, wasteland, and barren hills. In 2016, the government implemented a construction project of 10,000 mu, and the ecological restoration effect gradually manifested. As a result, the green wealth sub-index gradually rebounded to 1.1 in 2019.

#### Regional economic factors and green development

The “green growth” subsystem and its indicators were used to characterize the elements of regional economic development. As illustrated in Fig 2, the green growth index showed a fluctuating upward trend from 2010 to 2019. The maximum projection value of the green growth subsystem was 0.73, while the parameters with great impact on the development of the subsystem included proportion of resident energy consumption (0.67), energy consumption (t standard coal) (0.45), annual output value of the tertiary industry (0.35), annual output value of the secondary industry (0.29), land output rate (0.28), and output value of the primary industry (0.24). Energy consumption was negatively correlated with local economic development, while the others were positively correlated. With China’s rapid economic growth and urbanization, the total domestic energy consumption of Chinese residents showed an upward trend, accounting for 13% of the total energy consumption, which is lower than the average world level of 31% [45], while the maximum proportion of residents’ energy consumption in the study area was 20.3%, also showing a gradual upward trend. This reveals that energy conservation by residents in this region should be one of the key focal points of energy conservation during the 14^th^ Five-Year-Plan period and that local governments must advocate for residents’ energy conservation, green consumption, and living a low-carbon lifestyle [17].

Given the positively related parameters affecting regional green growth under the current shortage of land resources, making rational use of land, improving land use efficiency, and realizing the sustainable development of the regional economy, society, and ecology are critical issues for local government.

The rate of land output is a comprehensive economic index indicating the level of agricultural productivity in a region. In contrast, urbanization development has an agglomeration and spatial spillover effect on local production factors, resulting in synergy and integration between local industries and enterprises, improved input–output efficiency, and gradual optimization and upgrading of industrial structure. With the continuous integration and development of local primary, secondary, and tertiary industries, the comprehensive development quality and cooperation of regional land and resources have been improved. Additionally, industrial activity was the main behavior of the regional system and human relations, and all pollutants control center, the important carrier of the ecological environment and economy [6, 16]. The combination of resources, environment, and economy largely determine the degree and type of resource utilization and economic benefits in the region and the load on the ecological environment system. Under the background of green development and ecological civilization construction, the adjustment and optimal allocation of the local industrial structure are the key points to breaking the resource and environmental constraints and realizing the coordination between humans and land.

#### Elements of local social development and green development

While measuring the green development coefficient of the ecological project construction area in Baoshan, the “green welfare” parameter system was adopted to measure the interaction and influence between the local social system elements and green development. The overall development of the green welfare subsystem shows a fluctuating rising state during 2010–2019. The optimal projection value was 0.959, the highest projection value among the three subsystems and the most abundant representation of the green development index.

The main influencing parameters were as follows: Urban population (0.427), annual per capita disposable income of urban residents (0.424), per capita occupied public green space area (0.406), per capita net income of farmers (0.331), number of population (0.32), effective irrigated area (0.319), rural electricity consumption *(0.284), and “urban green land rate (0.258). Parameters were positively correlated with the development of green welfare subsystem, with the exception of rural electricity consumption. Urbanization increases the degree of population aggregation and provides a sufficient labor force in primary, secondary, and tertiary industries. The income of urban residents and the demand and desire for eco-environmental quality will force the government to pay close attention to local resources and environmental problems [30]. This can improve and enhance environmental quality, attract high-quality human resources, and enhance the residents’ awareness of the ecological ethos which fosters the formation of an economic development model of local endogenous growth. In contrast, the larger population density in the region places pressure on the utilization of public resources and environmental quality. For instance, the increase in electricity consumption and food demand will reduce the per capita resource ownership in the region as well as the ecological space, aggravating the situation of regional resources and the environment.

The combined ecological project construction of 10,000 mu improves regional human settlement environment quality. The sensitive ecological areas within 123.3 km^2^ of Baoshan were strictly protected and restored with 247,500 m^2^ being new green areas, 37.23% of the built-up areas are covered by green areas, while 98.4% of the central urban area has excellent air quality throughout the year. A total of 460 million yuan (68.08 million US$) was invested in repairing 92,000 m^2^ of damaged roads and sidewalks and dredging 36 heavily polluted rivers. The living environment in the urban and rural areas has been dramatically improved.

## Discussion

Green development has been proposed to tackle the increasingly severe resource and environmental problems and change the current development mode. With industrialization and urbanization, western scholars first studied green development based on the developmental path and the concrete implementation method of exploration [6, 19]. Meanwhile, domestic research on green development has emerged relatively recently, mainly focusing on developing a green concept, connotation, necessity, and national or provincial level of green development or green economic efficiency. However, the green economy is only one aspect of green development, which includes the green economy, social progress, development, and evolution. It focuses on the coordinated and sustainable development of the economy and the natural ecosystem.

Under the theoretical framework of regional green development, this study analyzed the green development evolution, trends, and influencing factors of 10.000 mu of ecological engineering construction area in Baoshan, Yunnan Province, using econometric analysis methods such as the PPM and gray system prediction model.

1. The theoretical framework of regional green development is based on the interaction and coordination mechanism of an ecological, economic, and social complex system of sustainable development from the perspective of system science. Green development promotes the harmonious development between humans and land (as well as nature) and the continuous optimization of its development model [15, 28]. Green development covers three subsystems, namely green wealth, green increase, and green welfare. Green wealth is the foundation of local green development construction, green growth is the core of regional economic development, and green welfare is the goal of the regional green development system. The coordinated and balanced development of the three subsystems is the intrinsic requirement for green development.
2. For the temporal evolution of green development, the overall system index of green development in Baoshan showed an upward trend, increasing from 1.31 in 2010 to 3.71 in 2019, with an average annual growth rate of 18.3%. The temporal evolution of each subsystem showed that the development trend of green wealth and development and the other subsystem was dislocated, indicating that the economic and social development of the study area was strongly linked to the development and utilization of ecological resources and the environment.
3. The factors that affect the regional level of green development were characterized by locality, dynamism, and complexity. Local urbanization, industrial structure, population, environment, and resources influence regional green development through economic ecology, land resource development, and the input–output mechanism.

Green development has become a mainstream global development trend; countries are exploring and practicing green development paths, gradually obtaining their own green development experiences. In the United States, green development focuses on the green energy field and promotes the transformation of traditional highly polluting high energy-consuming industries to clean energy through economic, scientific, technological, and legislative measures; the United Kingdom adopted measures such as environmental pollution control, industrial transformation, and legislation to transform into a clean environment and green and livable international region; Germany increased investment in green science, technology research, and development, improved the legal system of environmental protection, and promoted environmentally friendly products with its current global market share reaching 20% [46]. Singapore introduced the “Singapore 2030 Green Development Blueprint” to create a livable environment based on transportation, economy, infrastructure, and combating climate change; Japan vigorously developed clean energy, recycling, and environmental education for all people; France constructed green cities and piloted green development, focusing on construction and evaluation of a green city [14], as well as planning and development of sponge cities, smart cities.

By studying the dynamic time series evolution and impact of green development in Longyang District, Baoshan City, Yunnan Province, China, this study further revealed that other regions must also focus on the synergy between “development” and “green” to improve the overall level of regional green development from a systemic perspective. Each region should practice differentiated management and environmental treatment in line with its ecological, economic, and social development and promote green industrial development and clean production with the goal to save energy and reduce consumption and pollution [47]. The green development of cities in different regions shall be closely monitored at the national level, and policy support shall be given to effectively narrow the gap between the economic development and industrial structure of each region. In addition, inter-regional cooperation shall be guided to achieve complementarity and mutual reinforcement. Each region gradually forming appropriate strategies according to its resource and environment bearing capacity, existing economic development intensity, and development potential [14]. Regions with a higher level of green development are hot spots that radiate toward surrounding areas to fuel positive interactions and coordinated development across the region.

## Conclusions

Based on the requirements of green development in China and regional ecological-economic-social development, we arrived at the following concluding suggestions:

1. The concept of Chinese ecological civilization and green development was firmly established, aiding comprehensive decision-making for local sustainable development. The concept of “GDP only” should be gradually adapted, and the coordinated development of the man–land system should be internalized into the development planning and decision-making of local governments, the production practice of enterprises, and the whole process of life and consumption of the public [5, 6]. Strengthening green and economic lifestyles further and incorporating ecological civilization education into the teaching content of local colleges, middle schools, and primary schools will be a primary goal.
2. Improving the local ecological, economic development, and the quality of economic development. Considering economic ecology to be the goal, explore the green, low-carbon, circular and clean development path of the traditional industrial economy, promote the formation of a circular development system of enterprises, parks, industries, and regions, gradually forming a circular development system and integrated management mechanism [13]. Considering agricultural supply-side structural reform to be the core, ecological engineering construction projects as the main carrier, and innovate the main business model. Based on the industry of “vegetable, flower, and fruit,” the company innovates three business cards of flower, fruit and vegetable cloud, and 13,000 mu of vegetables, flowers, and fruits have been planted. Meanwhile, jointly create smart agricultural science and technology demonstration zone including modern high value-added agriculture, the future of agriculture and incubator, balanced nutrition and health experience, big data, Internet and electronic commerce, and public welfare and education, five new modules, which realize great achievement for the development of modern new agriculture. Accelerate the transfer of surplus land for 10,000 mu of agricultural ecological tourism zones; involved in modern enterprises such as the East Garden, Huada Genetics Group, and Baonong Science and Technology; to develop new types of economic entities, such as professional cooperatives, individual contractors, and mixed joint-stock investment enterprises, to develop large-scale order agriculture, provide farmers with all-round technical training, and make modern agriculture more standardized, refined, and efficient.
3. Improve the environmental management system, the carrying capacity of the regional ecological environment, and the quality of living environment and happiness. Implement the control of regional environmental and ecological risk processes and the protection and restoration supervision of mountains, water, forests, grass, fields, and lakes, improving the zoning scheme of ecological functions. To formulate local development plans for resource conservation and recycling, promote green energy conservation and gradient utilization of energy in local buildings and transportation [11, 25]. Guided by the market construction of resource and environment property rights, ecological compensation system and environmental economic system should be established to reflect the cost of resource, environment depletion, and ecological restoration. Supervise the disordered expansion of development areas, gradually optimize the production-life-ecology space, and improve the benefits of regional sustainable development; build a system framework that integrates regional urbanization, population, industrial structure, market, resources, environment, and other factors to drive the dynamic evolution of green development.

## Supporting information

S1 File (ZIP)

## References

[1] Guo FY, Lyu X, Yu W, Ren JM, Chu NC (2020) Performance evaluation and driving mechanism of green development in Shangdong province based on panel data of 17 cities. Sci Geogr Sin 40(2): 200–210.

[2] Hu AG, Tang X (2015) Green Development: The sustainable road of China’s development. National situation reports 18: 264–272.

[3] Long RY, Shao TX, Chen H (2016) Spatial econometric analysis of China’s province level industrial carbon productivity and its influencing factors. Applied Energy 166: 210–219.

[4] Chu DJ (2012) New concept of Green Economy and consideration of deepening Green Economy studies in China. China Population. Resource and Environment 22(5): 40–47.

[5] Mu XY, Liu K, Ren JL (2017) Spatial differentiation and change of green production efficiency in China. Progress in Geography 36(8): 1006–1014.

[6] Li XW, Du JG, Long HY (2018) A Comparative study of Chinese and foreign green development from the perspective of mapping knowledge domains. Sustainability 10: 1–30.

[7] Cabernard L, Pfister S (2020) A high resolved MRIO database for analyzing environmental footprints and green economy progress. Sci Total Environ 755: 142587. 33268260 10.1016/j.scitotenv.2020.142587

[8] Zhou L, Zhou CH, Chen L, Wang B(2020) Spatio-temporal evolution and influence factors of urban green development efficiency in China. J Geogr Sci 30(5): 724–742.

[9] Halkos G, de Alba J M, Todorov V(2020) Economies’ inclusive and green industrial performance: An evidence based proposed index. Cleaner Production 279(2): 123516.

[10] Tseng ML, Islam S, Karia N, Fauzi FA, Afrin S(2019) A literature review on green supply chain management: trends and future challenges resources. Conser Recycl 141: 145–162.

[11] Gan VJL, Lo IMC, Ma J, Tse KT, Cheng JC, Chan CM(2020) Simulation optimization towards energy efficient green buildings: current status and future trends. Journal of Cleaner Production 254: 120012.

[12] Gao Y, Yang GS, Xie QH (2020) Spatial-Temproal Evolution and Driving Factors of Green Building Development in China. Sustainability 12(2773): 1–21.35136666

[13] Lu CY, Cheng W, Huang P (2022) Spatio-Temporal comprehensive measurement of China’s Industrial green development level and associated influencing factors. Ecological Economy 38(3): 54–69.

[14] Zhang YL, Wu XL (2022) Urban green development level and spatio -temporal difference of cities in the National Key Ecological Function Zone and adjacent non-ecological function zones. Acta Ecological Sinica. 42(14): 1–17.

[15] Cheng Y, Wang JJ, Wang YP, Ren J (2019) A comparative research of the apstial-temporal evolution track and influence mechanism of green development in China. Geogr Res 38(11): 1–21.

[16] Peng BH, Zhang XC, Ehsan E, Wan AX (2022) Evolution of spatial-temporal characteristics and financial development as an influencing factor of green ecology. Environ Dev Sustain 24: 789–809.

[17] Xie ZX, Qin YC, Shen W, Rong PJ (2017) Efficiency and impact factors of low carbon economic development in China. ECONOMIC GEOGRAPHY 37(3): 1–9.

[18] Sun CZ, Tong YL, Shen W (2018) The evolution and a temporal spatial difference analysis of green development in China. Sustainable Cities and Society 41: 52–61.

[19] Hu AG, Zhou SJ (2014) Green Development: functional definition, mechanism analysis and development strategy. China Population, Resource and Environment 24(1): 14–20.

[20] Gao Y, Mu HK, Zhang YL, Tian Y, Tang DW, Li X (2019) Research on construction path optimization of urban-scale green network system based on MSPA analysis method: Taking Zhaoyuan City as an example. ACTA ECOLOGICAL SINICA 39(20): 7547–7556.

[21] Wei JX, Song Y, Wang YC, Xiang WN (2019) Urban green infrastructure building for sustainability in areas of rapid urbanization based on evaluating spatial priority: a case study of Pukou in China. ACTA ECOLOGICAL SINICA 39(4): 1178–1188.

[22] Zhao Haixia, Wang SF, Meng F, Niu MJ, Luo XL (2020) Green space pattern changes and its driving mechanism: a case study of Nanjing. ACTA ECOLOGICAL SINICA 40(21): 7861–7872.

[23] Hou CG, Ren JL, Cheng Y, Liu SF (2021) Spatial evolution and driving mechanism of China’s greenization process. Scientia Geographica Sinica 38(10): 1589–1596.

[24] Zhao L, Gao XT, Liu YX, Han ZL (2021) Evolution characteristics of spatial correlation network of inclusive green efficient in China. ECONOMIC GEOGRAPHY 41(9): 69–90.

[25] Sun HP, Edziah BK, Sun CW, Kporsu AK (2019) Institutional quality, green innovation and energy efficiency. Energy Policy 135: 111002.

[26] Tang YK, Wang K, Ji XM, Xu H, Xiao YQ (2021) Assessment and Spatial-Temporal Evolution Analysis of Urban Use Efficiency under Green Development Orientation: Case of the Yangtze River Delta Urban Agglomerations. Land 10(715): 1–19.

[27] Che L, Bai YP, Zhou L (2018) Spatial pattern and spillover effects of green development efficiency in China. Sci Geographica Sinica 38(11): 1788–1798.

[28] Wang Y, Li HY, Yu H (2018) Analysis of spatial pattern and evolution characteristics of provincial green development in China. China Population, Resources and Environment 28(10): 96–104.

[29] Du JG, Zhang J, Li XW (2020) What Is the Mechanism of Resource Dependence and High-Quality Economic Development? An Empirical Test from China. Sustainability 12(8144): 1–17.35136666

[30] Li XW, Du JG, Long HY (2019) Theoretical framework and formation mechanism of the green development system model in China. Environment Development 32: 1–13.

[31] Liu MG (2017) Chinese provincial green development level measurement and space evolution. Journal of outh China normal university (social science edition) 03: 37–44.

[32] Lorek S, Spangenberg JH (2014) Sustainable consumption within a sustainable economy: Beyond green growth and green economies. Journal of Cleaner Production 63: 33–44.

[33] Loiseau E, Saikku L, Antikainen R, Droste N, Hansjürgens B, Pitkänen K(2016) Green economy and related concepts: An overview. Journal of Cleaner Production 139: 361–371.

[34] D’Amato D, Droste N, Allen B, Kettunen M, Lähtinen K, Korhonen J(2017) Green, circular, bio economy: A comparative analysis of sustainability avenues. Journal of Cleaner Production 168: 716–734.36.

[35] Guo FY, Tong LJ, Qiu FD, Li YM (2021) Spatio-temporal differentiation characteristics and influencing factors of green development in the eco-economic corridor of the Yellow River basin. Acta Geographica Sinica 3: 63–65.

[36] Carfi D, Schiliro D (2012) A competitive model for the green economy. Economic Modeling 29(4): 1215–1219.

[37] Li L, Chen BP (2012) Ecological footprint and Green Development in China. China Population, Resouces and Environment 22(5): 63–65.

[38] Li ZL, Luo XF, Zhang JB (2016) Green economy of agriculture and its spatial convergence in China based on energy analytic approach. China Population, Resources and Environment 26(11): 150–159.

[39] Li XX, Liu YM, Song T (2014) Measurement of the Human Green Development Index. Social Sciences in China 35(6): 69–95, 207–208.

[40] Shen XY, Wang GH, Huang XJ (2017) Green GDP Accounting and Spatio-temporal Pattern in China from 1997–2013. Journal of Nature Resources 32(10): 1639–1650.

[41] Qian ZM, Liu XC (2014) A study of Regional Differences and Convergence of Green Economic Efficiency in China. Journal of Xiamen University (Arts & Social Sciences) 89(1): 110–118.

[42] Yang DG, Gao FP(2006) Theory analysis of Green Development road. Scientific management research (05): 20–23.

[43] Xiang SJ, Zheng RK (2012) Study on the green economy development index in China. Statistical Research 3: 72–77.

[44] Liu SF, Zheng B, Liu JF, Xie NM (2014) Several basic models of GM (1,1) and their applicable bound. Systems engineering and electronics 36(3): 501–508.

[45] Yang J, Khanna N, Li PC (2020) The analysis of situation and trend of Chinese residential energy consumption. Energy of China 42(12): 8–13.

[46] Wang WC (2022) Some thoughts on Green Development in urban construction. Construction Science and Technology (12): 84–87.

[47] Yang XM, Huang HP, Zhou RH (2023) Evaluation and spatiotemporal evolution of urban green development level in China. ACTA ECOLOGICA SINICA 43(4): 1–13.

