# Correction: Research on the temporal evolution track and influence of green development from 2010 to 2019

**DOI:** 10.1371/journal.pone.0314375

**Published:** 2024-11-20

**Authors:** Yunfang Chen, Xuan Ji, Qingkui Lai

There is an error in affiliation 2 for author Xuan Ji. The correct affiliation 2 is: Yunnan Key Laboratory of International Rivers and Transboundary Eco-Security, Yunnan University, Kunming, China.
